# RGO/CuCl-Based Flexible Gas Sensor for High-Concentration Carbon Monoxide Gas Detection at Room Temperature

**DOI:** 10.3390/mi15060737

**Published:** 2024-05-31

**Authors:** Qingqing Liu, Fuzheng Zhang, Mengfei Pei, Weile Jiang

**Affiliations:** 1 School of Humanities and Social Science, Xi’an Jiaotong University, Xi’an 710049, China; qing_831@xjtu.edu.cn (Q.L.); jiangwl@xjtu.edu.cn (W.J.); 2 State Key Laboratory for Manufacturing Systems Engineering, Xi’an Jiaotong University, Xi’an 710049, China; 3 Shandong Laboratory of Yantai Advanced Materials and Green Manufacturing, Yantai 265503, China; 4 Department of Heritage Management, Emperor Qinshihuang’s Mausoleum Site Museum, Xi’an 710600, China; vicky_masaki1103@163.com; 5 Joint School of Designed and Invovation, Xi’an Jiaotong University, Xi’an 710049, China

**Keywords:** flexible gas sensor, carbon monoxide, room temperature detection, composite film

## Abstract

Carbon monoxide (CO) gas sensors are widely used, especially for environmental monitoring in confined spaces such as the landscape of mining cave ruins in mining parks, which is essential for ensuring the health and safety of tourists and staff. In this paper, a flexible CO gas sensor based on polyimide, interdigital electrodes, and reduced graphene oxide (RGO)/cuprous chloride (CuCl) composite film is designed and manufactured for reliable room temperature detection of high-concentration CO gas. The structure size of RGO/CuCl gas-sensitive film is 5 × 5 mm. The RGO with a 62.65% C-C bond is prepared by the thermal reduction method. The test results show that the sensor has a high response in the range of 400–2000 ppm CO gas concentration, and the maximum response is 1.56. The linear correlation coefficient of the sensor is 0.981, which indicates that the sensor has good output response characteristics. The response time of the sensor for 400 ppm CO gas is 332 s, which indicates that the sensor has a fast response rate. Furthermore, compared with other gases, the sensor shows higher gas selectivity for CO gas. This sensor has the characteristics of small size and easy attachment; therefore, it can be installed on the shoulder or helmet of tourists’ safety suits, providing personalized real-time warning prompts for tourists’ physical health status.

## 1. Introduction

Carbon monoxide (CO), a colorless and odorless gas, combines with hemoglobin to form carboxyhemoglobin, which is unable to provide oxygen to the body’s tissues and causes oxygen deprivation in the blood [1]. When the CO gas concentration in the environment is 667 ppm, about half of the hemoglobin in the human body will be converted to carboxyhemoglobin, which seriously endangers human life. If the CO gas concentration reaches 2000 ppm, healthy adults will feel headaches, dizziness, and nausea within 20 min and will die within 1 h [2]. Because CO gas is colorless and odorless, it is easy to be ignored in daily life, which has great safety risks.

In recent years, the landscape of mining cave ruins in mining parks has been deeply loved by tourists. After the restoration of the geological environment in the mine, the original abandoned mining cave space is used to display geological relics and mining production activity relics, which can be a specific landscape for people to visit. Due to the need for an authentic experience, the surface of the mining cave is mostly still exposed to natural minerals. Large exposed ores such as coal mine stones may produce harmful gases such as CO after natural long-term oxidation, especially in this type of mining cave space, which is confined, deep underground, relatively closed, generally with few entrances and exits, and has lower oxygen content than the surface. If the ventilation conditions inside the mine are limited, once the accumulation of CO gas exceeds the limit, it will seriously threaten the safety of tourists and staff. Factors such as age, health status, and personal physiological characteristics lead to differences in individual sensitivity and tolerance to CO gas. Therefore, it is necessary to design a portable real-time warning and detection sensor for tourists in such heritage parks. In addition, although this kind of place is underground, in order to protect the ruins from moisture damage and provide a good environment for tourists, a large number of dehumidifiers are used to make their internal environment relatively dry.

How to timely and conveniently detect high-concentration CO gas in the confined space of mining cave sites and reduce the risk of CO poisoning has become an important topic in the monitoring, warning, and emergency rescue at such heritage parks. It also brings greater opportunities and challenges for the research and development of personalized and convenient CO gas sensors. Traditional gas sensors are usually manufactured on inorganic substrates, including quartz, glass, alumina ceramic tubes, etc. [3,4]. There are widespread problems such as high operating temperature, high power consumption, poor resistance to bending and stretching, and especially, the gas sensing characteristics of the sensor at room temperature are poor or even unable to work, which seriously limits the development and application of gas sensors in the field of low-power wearable electronics [5]. Steinhauer et al. [6] proposed a novel gas sensing device based on cupric oxide (CuO) nanowires synthesized on-chip by thermal oxidation of electroplated copper microstructures. The sensor has a response of 1.2 (R_a_/R_g_) to 100 ppm CO gas at 300 °C. Lin et al. [7] used Cu nanowires to synthesize CuO nanotubes for CO gas detection through a high-temperature annealing process, which has a response of 1.13 (R_a_/R_g_) to 100 ppm CO gas at 100 °C. Compared with other gas sensors with rigid structures, flexible gas sensors manufactured on soft substrates such as polymer, textile, and paper substrates overcome the disadvantages of large volume, poor ductility, and low flexibility of traditional rigid structures [8,9]. Therefore, they are more suitable for complex situation monitoring and fine operation requirements, which have shown great application prospects and value in the consumer electronics market, such as in wearable devices and smart mobile terminals [10,11]. Zhang et al. [12] designed and developed a flexible CO sensor based on interdigital electrodes and SWCNTs/Pt composite film. The sensor enables reliable detection of 0–100 ppm CO gas at room temperature with a sensitivity and linearity of 0.0201 Ω/ppm and 94.5%, respectively. Based on the single-walled carbon nanotube (SWCNT), Zhang et al. [13] proposed a flexible gas sensor for the detection of CO at room temperature, which has a room temperature response of 1.2 (R_a_/R_g_) to 100 ppm CO gas. Although there is much research on flexible CO gas sensors, there are still many problems such as complex structure and preparation process, narrow detection range, and low detection upper limit. Therefore, the development of a flexible high-concentration CO gas sensor that can work reliably at room temperature is an urgent problem and focus.

In order to further improve the gas-sensitive performance of gas sensors, the current mainstream method is to functionalize gas-sensitive materials through nanoparticle doping, surface modification, and other means, such as the use of dopants for surface structure regulation, noble metal nanoparticles modification, heterojunction construction, etc., which has been proven to be a scientific and effective method [14,15]. Graphene [16] is the hardest nanomaterial known to date and exhibits excellent mechanical properties, which theoretically have a Young’s modulus and strength of about 1 TPa and 130 GPa, respectively. Another important property of graphene is that it can be functionalized with functional groups through covalent or non-covalent interaction, especially plasma oxidation, graphene oxide reduction, etc., and can produce a large number of hydroxyl, carboxyl and other oxygen-containing functional groups on its surface or edge, which can effectively change the physical and chemical properties of graphene [17]. Graphene has the advantages of a large specific surface area, excellent electrical and mechanical properties, and rich surface chemistry and electrocatalytic activity, and is widely used in biomedicine, energy storage and conversion, photocatalysis, and sensors [18]. Cuprous chloride (CuCl) is a tetrahedral white crystal that gradually oxidizes to green basic CuCl when exposed to air but breaks down again when exposed to light. In general, it is stable in dry air, but is prone to moisture and turns blue. Moreover, it can react with CO gas molecules in the air and is widely used to detect CO concentration in mixed gases [19]. Therefore, given the excellent physicochemical properties of graphene and CuCl, an excellent gas-sensitive material for the detection of CO gas at room temperature can be obtained by doping graphene with CuCl nanomaterial. Compared with other oxides and 2D materials, the advantages of this RGO/CuCl composite film mainly include two aspects: one is a large specific surface area and excellent electron transfer rate; the second is that CuCl can form a complex with CO gas, which further enhances its gas-sensitive properties and selectivity to CO gas. The disadvantage is that the composite film is easily affected by environmental humidity, so the best working environment is a dry air environment.

Briefly, aiming at the requirements of room temperature CO detection and wearable application of a CO gas sensor, a flexible CO gas sensor based on a micro-nano manufacturing process and an RGO/CuCl composite film structure is designed and presented in this paper. The RGO nanomaterial is successfully prepared by the thermal reduction method, that is, solid graphite oxide is produced by an improved Hummers method and then reduced to RGO after heat treatment. In addition, the morphology and reduction degree of the prepared RGO are also characterized by SEM, Raman, XRD, XPS, etc. Finally, the response characteristics of the sensor to high-concentration CO gas are tested at room temperature, and the room temperature response and sensitization mechanism of the sensor are analyzed.

## 2. Structural Design and Preparation of the Sensor

**Figure 1:**
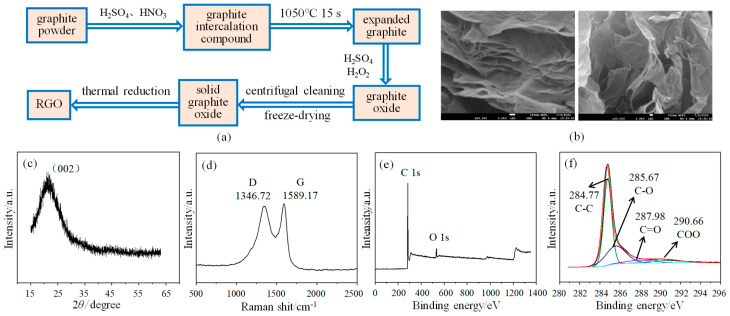
Preparation and characterization of RGO: (**a**) Preparation process; (**b**) Surface topography; (**c**) XRD spectrogram; (**d**) Raman spectrogram; (**e**) Full spectrogram of XPS; (**f**) C1s spectrogram of XPS.

The sensor consists of a polyimide substrate, an interdigital electrode, and an RGO/CuCl composite film, in which RGO is prepared by an improved Hummers method [20]. The good flexibility of the PI substrate gives the sensor better resistance to bending and stretching. As shown in Figure 1a, the expanded graphite is first obtained by oxidizing agent and high-temperature heat treatment, and then graphite oxide is obtained after treatment with concentrated sulfuric acid and hydrogen peroxide. The solid graphite oxide is made by the centrifugal cleaning and freeze-drying of graphite oxide. Finally, the solid graphite oxide is placed in a tubular annealing furnace and subjected to high-temperature heat treatment at 750 °C to obtain the RGO. N_2_ is used as a protective gas and the heat treatment time is 2 h. The surface topography of RGO is shown in Figure 1b. It can be seen that the structure of RGO is thin and stacked together, and also has a certain state of curling and folding, which shows the typical characteristics of two-dimensional materials. X-ray diffraction (XRD) is used for the structural analysis and characterization of materials. RGO has a relatively wide diffraction peak (002) near 2θ = 22° (See Figure 1c), which is consistent with the standard card (JCPDS no. 41-1487) data and confirms the successful synthesis of RGO [21]. In addition, the wide characteristic peak also indicates that the RGO produced after reduction still has many defects, and its degree of order is not very high. As shown in Figure 1d, the analysis results of the RGO Raman spectrum show that there are two obvious characteristic peaks near 1346.72 cm^−1^ and 1589.17 cm^−1^, respectively: D-peak and G-peak [22]. I_D_ and I_G_ represent the area corresponding to peak D and peak G, respectively, and the ratio I_D_/I_G_ represents the defect and disorder state of RGO. The larger the ratio, the more defects there are. The ratio of I_D_/I_G_ is calculated to be 1.94, indicating that there are still many defect structures, such as hydroxyl and epoxy groups, in RGO after reduction, which provides many active sites for gas adsorption and is conducive to its application in the field of gas sensing. X-ray photoelectron spectroscopy (XPS) enables qualitative and quantitative analysis of materials’ elemental composition and chemical structure of materials. As shown in Figure 1e, only the characteristic peaks corresponding to the C and O elements are detected, which shows that no other impurities are introduced into RGO during its preparation [23]. Figure 1f shows the C1s spectrum of the RGO, in which four peaks centered on 290.66 eV, 287.98 eV, 285.67 eV, and 284.77 eV are observed, corresponding to four types of carbon bonds: COO, C=O, C-O, and C-C, respectively [24]. Among them, the proportion of the C-C bond is the largest at 62.65%, indicating that RGO has been successfully prepared by the thermal reduction method.

**Figure 2:**
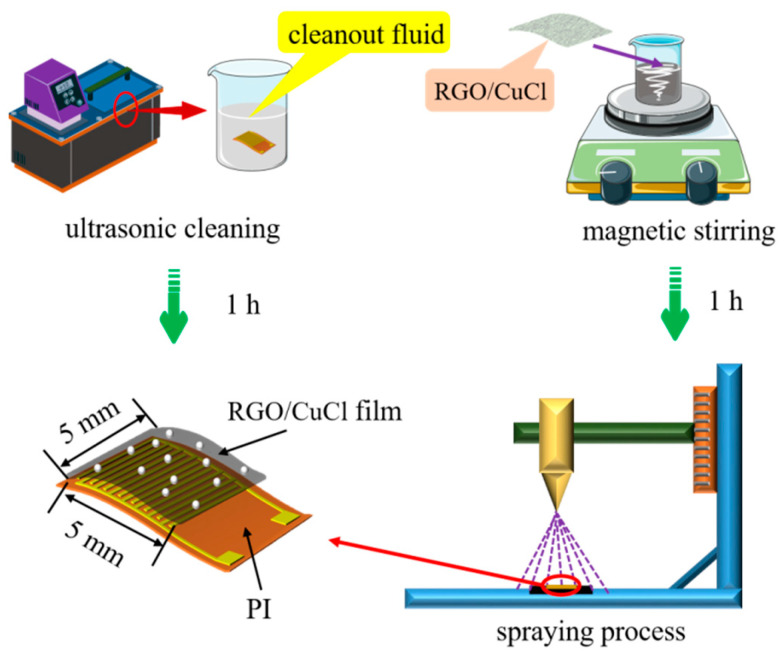
Preparation of the RGO/CuCl-based sensor.

The flexible interdigital electrode substrate is prepared by a micro-nano manufacturing process, in which the substrate size is 7 × 11 mm and the area of the interdigital electrode is 5 × 5 mm. Cu with good conductivity is used as an interdigital electrode material. The preparation process of the sensor is shown in Figure 2. First, the interdigital electrode substrate is cleaned to remove impurities such as dust on its surface. Cleaning solutions include acetone solution, anhydrous ethanol, and deionized water. The cleaning time of each cleaning solution is 20 min. After cleaning is completed, the substrate surface moisture is blown dry by drying N_2_. Then, RGO/CuCl water dispersion is formulated as follows: (1) 5 mg of RGO powder is placed in a small beaker, then 10 mL of ultra-pure water is added to it, and the RGO is evenly dispersed in the aqueous solution by the magnetic stirring method, which finally obtains an RGO homogeneous aqueous dispersion with a mass fraction of 0.05 wt%; (2) Then, 10 mg CuCl nanopowder is added to the RGO water dispersion and it is dispersed evenly by the same method to finally obtain the RGO/CuCl water dispersion; (3) Finally, 0.25 mL RGO/CuCl water dispersion is used to form an RGO/CuCl composite film on the interdigital electrode substrate by a spraying process; then, the sensor is placed in a drying oven and dried in a constant temperature environment of 60 °C for 1 h. Compared with the drop coating and spin coating methods, the spaying method can attach the sensitive material aqueous solution to the sensor base in the form of mist through high-pressure gas, that is, the water in the solution is evaporated in this process, and only the sensitive particles are evenly dispersed on the base surface, which is more suitable for the film formation of a low viscosity solution.

**Figure 3:**
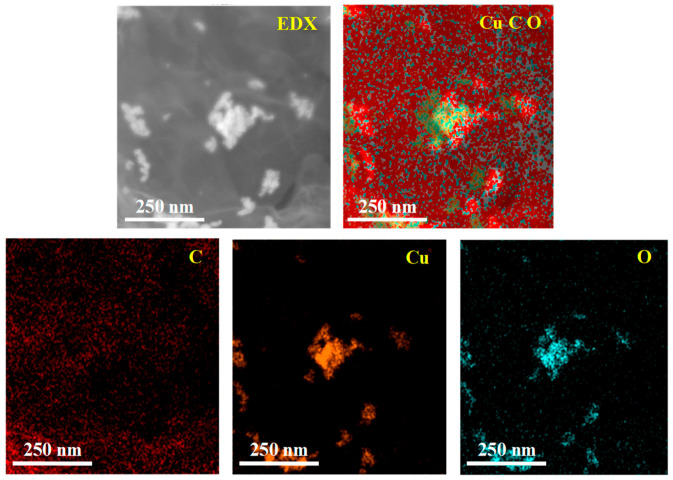
EDX characterization of RGO/CuCl composite film.

The energy dispersive X-ray (EDX) characterization of RGO/CuCl composite film is shown in Figure 3. The elements of C, O, and Cu are labeled as red, blue, and yellow, respectively, and the distribution area of the different colors indicates the content of each element. Because the Cu element exists only in CuCl nanoparticles, it can be seen from the Cu element spectrum that the yellow is CuCl nanoparticles and the black area represents RGO. CuCl nanoparticles are dispersed in RGO, which also proves the successful preparation of RGO/CuCl composite nanoparticles.

## 3. Experimental Result and Analysis

The gas sensor test platform uses dry air as a carrier gas to adjust different CO gas concentrations. The resistance change signal of the flexible CO gas sensor is collected by a digital multimeter (Keysight 34461A) and the collected data is stored on a computer through the corresponding software. The Keysight 34461A is purchased from Keysight Technology Co., LTD. (Santa Clara, CA, USA).The sensor’s test voltage is 5 V. The testing process of the sensor is as follows: First, air with a flow rate of 250 cm^3^/min (250 SCCM) is passed into the test chamber to eliminate other gases so that the sensor is in a completely clean and dry air atmosphere. After the resistance value of the sensor is basically stable, CO gas with a concentration of 400 ppm is passed into the test chamber by adjusting the gas distribution device. At this time, because the CO gas is adsorbed by the gas-sensitive film, the resistance value of the sensor will change. If the sensor’s resistance value tends to be stable, the CO gas is stopped and the air with a flow rate of 250 cm^3^/min is continued to be passed into the test chamber to desorb the CO gas from the gas-sensitive film. The performance test of the gas sensor is carried out at room temperature (20 °C). Different gas concentrations are adjusted by a mass flowmeter (Sevenstar CS200), which is a very mature and stable gas flow control system. The Sevenstar CS200 is purchased from Sevenstar Huachuang flowmeter Co., LTD. (Beijing, China).

**Figure 4:**
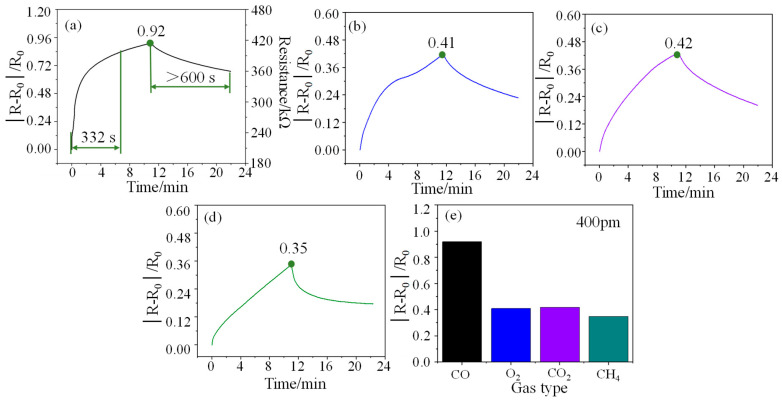
(**a**), (**b**), (**c**), and (**d**) are the response curves of the sensor to 400 ppm CO, O_2_, CO_2_, and CH_4_ gases respectively, and (**e**) is a comparison diagram of their responsivity.

Response refers to the relative change in the resistance of the sensor at a certain concentration, that is, the ratio of the change in the output resistance to the initial resistance (|*R* − *R*_0_|/*R*_0_). In a test cycle, the maximum response of the sensor is defined as responsivity (*S*), also known as sensitivity. The response curve of the sensor to 400 ppm CO is shown in Figure 4a. As can be seen from the figure, the response time and responsivity of the sensor for 400 ppm CO gas are 332 s and 0.92 at room temperature, respectively, which indicates that the sensor has a fast response rate and a high responsivity. However, the recovery performance of the sensor at room temperature is poor, exceeding 600 s. Furthermore, the initial resistance of the sensor is about 210 kΩ. The resistance change of the sensor at other concentrations can also be easily calculated through the responsivity formula. The response curves of the sensor for O_2_ (400 ppm), CO_2_ (400 ppm), and CH_4_ (400 ppm) gases are shown in Figure 4b–d, and their responsivity is 0.41, 0.42, and 0.35, respectively. Compared with other gases (See Figure 4e), the sensor shows higher gas selectivity for CO gas.

**Figure 5:**
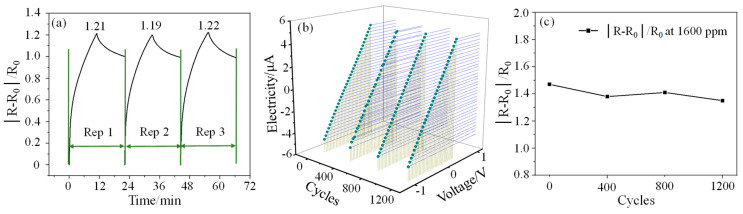
(**a**): Repeatability test results at 1000 ppm CO gas concentration; (**b**,**c**) are the I–V characteristic curves of the sensor and the response test results to 1600 ppm CO gas after different bending cycles, respectively.

The repeatability (Rep) testing of the sensor to 1000 ppm CO gas concentration is also carried out. As can be seen from Figure 5a, the responsivity of the sensor in the three cycles is 1.21, 1.19, and 1.22, respectively, and the overall change is small. Taking the first responsivity value as the benchmark, the second and third measurement errors are 1.7% and 0.83%, respectively, which indicates that the sensor has good responsivity consistency. However, it can also be found that the recovery of the sensor is relatively poor and cannot be completely restored to the initial state in a short period of time. The I–V characteristic curves of the sensor and the response test results to 1600 ppm CO gas after different bending cycles are shown in Figure 5b,c, respectively. The test results show that the current of the sensor has almost no change after different bending cycles, which indicates that the sensor has good electrical performance. The responsivity of the sensor to 1600 ppm CO gas after bending 400, 800, and 1200 times is 1.38, 1.41, and 1.35 respectively. Compared with the initial responsivity (1.47), their changes are all within 80%, showing good bending resistance.

**Figure 6:**
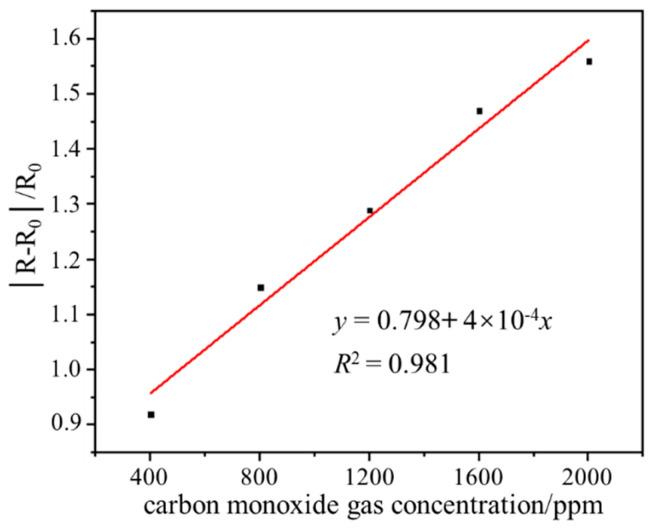
Gas sensing performance test result of the sensor in the range of 400–2000 ppm CO.

The gas sensing performance test result of the sensor in the range of 400–2000 ppm CO gas is shown in Figure 6. In the range of 400–2000 ppm CO gas concentration, the output responsivity of the sensor increases linearly with the increase of CO gas concentration, and the linear correlation coefficient is 0.981, which indicates that the sensor has good gas-sensitive response characteristics. The output responsivity of the sensor at 400, 800, 1200, 1600, and 2000 ppm CO gas is 0.92, 1.15, 1.29, 1.47, and 1.56, respectively. The wide range detection capability indicates that the sensor has the application value and potential to detect ultra-high-concentration CO gas leakage at room temperature.

**Figure 7:**
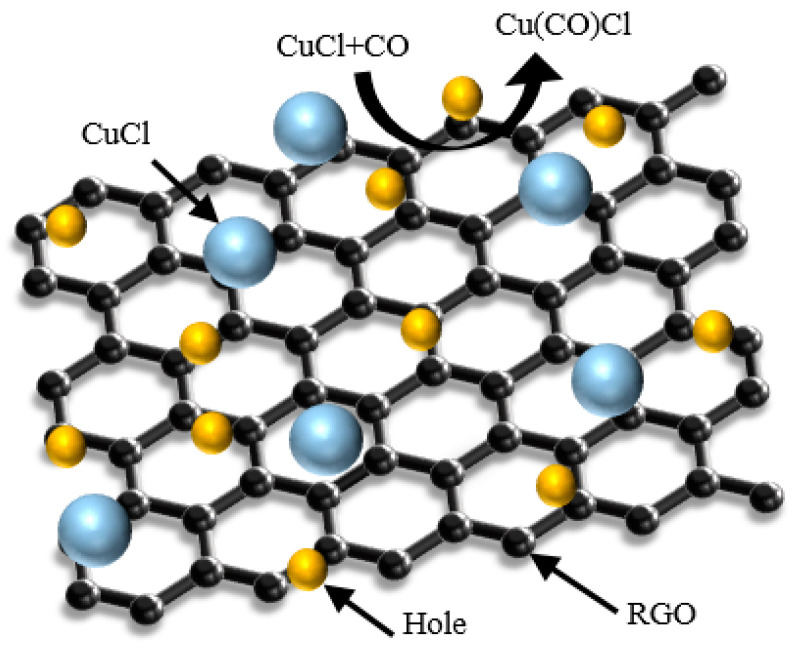
Schematic diagram of the gas sensing principle.

The schematic diagram of the gas sensing principle is shown in Figure 7. RGO is a P-type semiconductor that also acts as an acceptor or donor to accept or provide free electrons upon contact with the target gas, which causes a change in its own conductivity. CO is a reducing gas; after it is adsorbed by RGO, it will transfer a lone pair of electrons to RGO and bind with the holes in RGO to reduce the carrier concentration, which will lead to a decrease in the conductivity and an increase in the resistance of the sensor. In addition, inorganic salt CuCl nanoparticles are also uniformly dispersed around the RGO as doped particles, which also play a key role in the adsorption of CO gas. CuCl nanoparticles react with CO gas molecules to form a Cu(CO)Cl complex, which makes the sensor exhibit better sensitivity and gas-sensitive response characteristics. Therefore, the synergistic effect of RGO and CuCl nanoparticles enables the sensor to show a high gas-sensitive response to high-concentration CO gas at room temperature.

**Figure 8:**
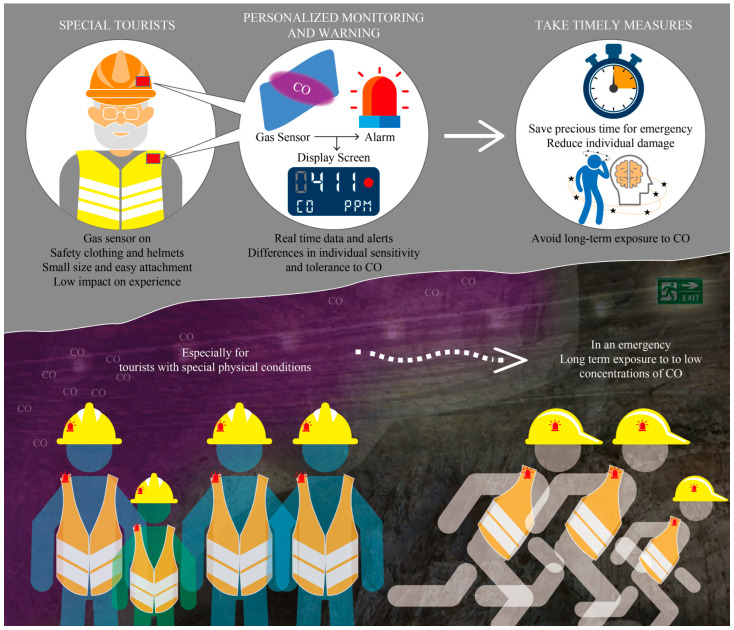
Application diagram of flexible CO gas sensor in the field of mine safety monitoring.

The application diagram of flexible CO gas sensors in the field of mine safety monitoring is shown in Figure 8. Based on the characteristics of low CO gas density, which is lighter than air, this CO gas sensor can be installed on the shoulder or helmet of the tourist safety suit, closest to the mouth or nose. Due to its small size and easy attachment, this sensor has a very low impact on the tourist experience. During the long journey of tourists, this sensor can detect the real-time concentration of CO gas and provide personalized prevention tips for each tourist’s health condition. At the same time, this sensor can also provide effective support for reasonable escape routes for tourists in sudden dangerous situations. In the future, this sensor can not only be used in the landscape of mining cave ruins but also in underground confined spaces with high traffic, such as railway tunnels and subways. The application prospects for detection, prevention, and emergency rescue are very broad.

## 4. Conclusions and Future Work

In summary, a flexible CO gas sensor for high-concentration CO gas detection is designed and proposed in this paper, consisting of a polyimide substrate, an interdigital electrode structure, and an RGO/CuCl composite film. RGO with a 62.65% C-C bond content is prepared by an improved Hummers method, and RGO/CuCl composite film is deposited on the interdigital electrode base by the spaying process. The response characteristic of the sensor to CO gas is tested at room temperature. The test results show that the sensor has a high output response and high linear correlation (0.981) to CO gas in the concentration range of 400–2000 ppm, among which the responsivity to 2000 ppm CO gas is 1.56 at room temperature. The sensor’s response time for 400 ppm CO gas is 332 s, which shows that the sensor has a fast response rate. However, the recovery of the sensor is poor, which is an issue that needs to be addressed in the future. In addition, the sensor is susceptible to humidity, so its stability is not ideal. This problem is also the biggest challenge facing this type of sensor, and it is also our main research direction in the future.

## Data Availability

The data that support the findings of this study are available from the corresponding author upon reasonable request.
